# Does pain mediate or moderate the relationship between physical activity and depressive symptoms in older people? Findings from The Irish Longitudinal Study on Ageing (TILDA)

**DOI:** 10.1080/21642850.2014.929006

**Published:** 2014-07-15

**Authors:** C. Kelleher, A. Hickey, R. Conroy, F. Doyle

**Affiliations:** ^a^ Division of Population Health Sciences, Royal College of Surgeons in Ireland, Dublin, Ireland

**Keywords:** depressive symptoms, mediation, moderation, older adults, pain, physical activity

## Abstract

*Background.* Depression is an increasing problem in older adults, which is exacerbated by under diagnosis and ineffective treatment options. Broadly speaking, as people age, their levels of regular physical activity (PA) decrease, while their experience of chronic pain increases. PA has been shown to be an effective, yet under-utilised, treatment for depression in this age-cohort although the influence of pain on the relationship between PA and depressive symptoms has not been considered. *Methods.* Secondary analysis of national data from The Irish Longitudinal Study on Ageing (TILDA, 2011) (*n* = 8163 participants aged 50 years and older) examined the mediating or moderating role of pain in the relationship between depressive symptoms and PA, and the impact of PA, pain and depressive symptoms on health-care utilisation. *Results.* Approximately 8.5% TILDA older adults were depressed. No mediating or moderating effects of pain were found in the association between PA and depressive symptoms. Higher levels of PA were found to be independently associated with lower depressive symptoms, while higher levels of pain significantly increased the likelihood of depressive symptoms supporting previous findings. Depressive symptoms and higher levels of pain were also found to significantly increase health-care utilisation. *Conclusions.* Consistent with previous findings in this field, both PA and pain were found to be independently associated with depressive symptoms in Irish older adults. Furthermore, pain does not play a mediating or moderating role in the relationship between PA and depressive symptoms. Continued support for ongoing initiatives in this area aimed at increasing PA in older adults as a means to improve both physical and mental well-being is advised. The absence of any synergistic effect between PA and pain suggests that clinicians and health service providers should continue to promote PA as a treatment for depression, irrespective of the pain levels of their patients.

## Introduction

By 2020, depression is projected to be the second leading cause of disease burden worldwide (Chapman & Perry, 2008). While estimates of depression rates largely focus on younger adults (i.e. <65 years), depression is also expected to disproportionately affect older adults (Heo, Murphy, Fontaine, Bruce, & Alexopoulos, 2008). This can be potentially accounted for by two trends. First, rates of depression in the general population are increasing (Compton, Conway, Stinson, & Grant, 2006). Second, the world's population is ageing with a predicted doubling in the proportion of people over the age of 60 years by the year 2050 (United Nations, 2002). In summary, future cohorts of older adults will display increased levels of depressive disorders than their predecessors (Chapman & Perry, 2008), suggesting that the need for effective and evidence-based interventions has never been more pressing. Furthermore, other established associations exist between depressive symptoms and a number of other sociodemographic factors. Higher levels of depressive symptoms have been demonstrated in women, non-married/cohabiting people, those of lower educational and socioeconomic groups, smokers and those with lower self-rated health (Cole & Dendukuri, 2003; Djernes, 2006; Everson, Maty, Lynch, & Kaplan, 2002; Morgan, O'Farrell, Doyle, & McGee, 2011; Zunzunegui et al., 2007). Therefore, any investigation of depressive symptoms in older people also needs to control for such factors.

Increased levels of depressive symptoms also have implications for the health-care services. Research suggests that depression in older adults is often undiagnosed and when it is, may be poorly treated (Lebowitz et al., 1997). High levels of medical comorbidity associated with depressive disorders also means that depressive symptoms can be disguised by physical complaints (Juurlink, Herrmann, Szalai, Kopp, & Redelmeier, 2004; Kales, Maixner, & Mellow, 2005; Oslin et al., 2002). This can make the diagnosis difficult and complicate the duration and effectiveness of treatment strategies (Chapman & Perry, 2008; Unützer, 2002). Furthermore, the provision of medical services is higher still for those with depressive symptoms and no formal diagnosis compared to those with a formal diagnosis (Johnson, Weissman, & Klerman, 1992). One large, multi-centre cross-national observational study (*N* = 18,849) examined the treatment of depression in primary health care across six countries. Across all centres, participants with depressive symptoms were twice as likely as those without such symptoms, to report three or more health-care visits in the previous three months (Herrman et al., 2002).

Physical activity (PA) has been identified as an effective but under-utilised treatment for depression (Dunn, Trivedi, Kampert, Clark, & Chambliss, 2005; Fox, 1999; Morgan et al., 2011). Furthermore, the positive effects of PA on physical health and well-being in older people are robustly supported in research (Allender, Hutchinson, & Foster, 2008; White, Wójciciki, & McAuley, 2009). For example, Giuli, Papa, Mocchegiani, and Marcellini (2012) surveyed a group of community-dwelling older adults (*N* = 306) about their levels of weekly exercise over the past year. They found that those who engaged in regular PA (≥1 hour of weekly exercise) were significantly more likely to have a lower body mass index, better self-rated health status and no symptoms of depression. National surveys (e.g. English Longitudinal Study of Ageing) have also demonstrated this link between increased levels of PA and lower levels of depressive symptoms (Banks, Nazroo, & Steptoe, 2012; Morgan et al., 2008; Morgan et al., 2011).

Relatively few studies have examined the role of pain in the association between PA and depressive symptoms. Sabiston, Brunet, and Burke's (2012) study examined pain, PA and symptoms of depression in female survivors of breast cancer (mean age = 54.9 years). They found a positive association between pain and depressive symptoms and a negative association between pain and PA. When this relationship was tested further, PA was found to partially mediate the relationship between pain and depressive symptoms (Sabiston et al., 2012). In another study, Mossey, Gallagher, and Tirumalasetti (2000) found that the effect of pain on physical functioning, in a group of elderly community-dwelling residents (*N* = 228), was a function of their level of depressive symptoms (i.e. an interaction effect was found). In summary, at all levels of pain, an increase in depressive symptoms was significantly associated with a higher probability of being in the lowest physical functioning quartile (Mossey et al., 2000). A recent study involving participants 50 years and older from Northern Ireland (NI) and the Republic of Ireland (RoI) (*N* = 6159) found core depressive symptoms (i.e. depressed mood and anhedonia) in 7.2% of their nationally representative sample (Morgan et al., 2011). This study also showed that those who were engaged in moderate to high levels of PA had a 50–56% reduction in the odds of having elevated depressive symptoms compared to those with low levels of PA (Morgan et al., 2011). Unfortunately, the analyses omitted a potentially significant explanatory variable – pain. Pain has been shown to be associated with increased risk for depressive symptoms in older persons (Bair, Robinson, Katon, & Kroenke, 2003; Onder et al., 2005), and is also a potential reason for non-engagement in PA (Mossey et al., 2000). It could therefore, interact, with (mediate or moderate the association between) depressive symptoms and PA. Additionally, both higher depressive symptoms and pain are associated with increased health-care utilisation (Blyth, March, Brnabic, & Cousins, 2004; Herrmann et al., 2002).

### The current study

The aims of this study were to investigate whether pain mediates or moderates the association between PA and depressive symptoms in Irish adults aged 50 years or more and secondly, to examine the effect of these variables on health-care utilisation. Mediators and moderators are usually third variables that facilitate a more in-depth understanding of the relationship between the variable of interest and the outcome measure (Wu & Zumbo, 2008). Mediation analysis explains the mechanism of how a variable operates via another (Frazier, Tix, & Barono, 2004). For example, it might be possible that the positive association between pain and depressive symptoms is explained by PA – higher pain leads to lower PA, which leads to higher depressive symptoms. In this case, PA would mediate the effect of pain on depressive symptoms. A moderation effect is also commonly known as an interaction effect where the strength of the effect of one variable (e.g. pain) on the outcome (depressive symptoms) varies with levels of another variable (PA) (Wu & Zumbo, 2008). For example, pain may not have an impact on depressive symptoms in those with high levels of PA, but it might for those with low or moderate levels of PA, above and beyond the effects seen for each variable alone. Moderation analysis, therefore, accounts for the ‘when’ and ‘for whom’, e.g. are the effects of the variable seen in outcomes for women but not for men (Frazier et al., 2004). In summary, the study objectives are:

- To profile the prevalence levels of depressive symptoms, PA and pain reported by older Irish adults.
- To ascertain any mediating and/or moderating effects in the relationships between these variables.
- To examine the impact of these variables on health-care utilisation.

## Methodology

The Irish Longitudinal Study on Ageing (TILDA, 2011) is a large-scale, nationally representative study of people aged 50 years and over living in the RoI conducted in 2010 (*n* = 8163) (Barrett, Savva, Timonen, & Kenny, 2011). The RANSAM system based on the Geodirectory developed by the Economic Social and Research Institute (ESRI) in Ireland (Whelan, 1979) was used to recruit participants. The sample design incorporated stratification, clustering and multi-stage selection (Kenny et al., 2010). The response rate was 62% and the final sample was weighted using estimates for age, sex and educational attainment from the Quarterly National Household Survey (QNHS, 2010). Further details on the survey methodology and sampling techniques used are available elsewhere (Barrett et al., 2011; Kenny et al., 2010).

### Outcome variables

*Depressive symptoms:* The primary outcome measure was the absence or presence of depressive symptoms as measured by the Centre for Epidemiological Studies Depression Scale (CES-D) (Radloff, 1977). This is a 20-item measure designed to measure symptoms of depression in the general population (i.e. non-psychiatric persons aged older than 18 years). Respondents rate the frequency of a range of depressive symptoms over the past week (e.g. depressive mood, loss of appetite, feelings of guilt and worthlessness). Radloff (1977) recommends a threshold of 16 to indicate a likelihood of clinically significant depression. Prevalence of depression was calculated using the threshold scores, i.e. presence of depression was indicated by a score of ≥16 on the CES-D. However, for chi-square, mediation and moderation analyses, a binary outcome variable (*z*-score) was created which classified participants as either ‘depressive symptoms’ or ‘no depressive symptoms’. All participants who scored 1 standard deviation (SD) or more above the mean were classified as ‘depressive symptoms’, while all remaining participants were classified as ‘no depressive symptoms’.

*Health-care utilisation:* The secondary outcome was *health-care utilisation*, measured by older adult's frequency of accessing general practitioner (GP) services in the last year. Participants were asked to indicate how many times they had seen their GP in the last 12 months. A four-level categorical variable was created representing: No GP visits; 1–2 GP visits; 3–4 GP visits and 5 or more GP visits in the last year.

### Independent variables

*Physical activity:* The validated short form of the International Physical Activity Questionnaire (IPAQ) (Craig et al., 2003) was used to assess levels of PA. The IPAQ includes a series of items that measure the length of time spent being physically active at different levels of intensity (e.g. from walking to vigorous exercise). The results were categorised into *low* (little or no PA, less than 5000 steps a day), *moderate* (approximately 5000–10,000 steps a day) or *high* (over 10,000 steps a day) rates of PA. *Pain:* Respondents were first asked ‘Are you often troubled with pain?’ Those who said ‘Yes’, they were then asked ‘How bad is the pain most of the time? Is it mild, moderate or severe?’

### Covariates

*Age and demographics:* The following age categories were created: 50–54 years; 55–64 years; 65–74 years and 75+ years for analyses. Other demographic covariates controlled for were gender, marital status, social class and education. *Self-rated health:* TILDA respondents rated their health as excellent, very good, good, fair or poor. Responses were recoded into a three-level categorical variable of self-rated health: poor; fair; good/excellent. *Smoking:* Respondents were asked if they had ever smoked cigarettes, cigars or cigarillos or a pipe daily for a period of at least one year. Those who answered ‘Yes’ to this question were then asked if they smoked at the present time (i.e. within 3 months of participation was classified as Yes). Respondents were categorised as being non-smokers, current smokers or former smokers/ex-smokers.

### Statistical analyses

Data were analysed using descriptive and inferential statistics. Analyses were carried out using the statistical package Stata Version 12 and Stata survey commands were used throughout as data were weighted and clustered. The overall level of missing data was low (less than 2% for all variables). Chi-square analyses explored differences between groups in demographic and health behaviour variables. All mediation and moderation analyses controlled for the effects of gender, age, marital status, social class, education, self-rated health, smoker status and number of GP visits in the last 12 months. All analyses were weighted as per the original data set.

Depressive symptoms (binary) was the primary outcome variable. Poisson regression was used to test for any mediating or moderating effect of pain on the association between PA and depressive symptoms, with risk ratios (RRs) used as the measure of effect size. First, in adjusted models, the independent relationship between PA and depressive symptoms was tested followed by another model that assessed the relationship between pain and depressive symptoms. Then, mediation effects were tested for by including both pain and PA in the same model. A substantial reduction in the RR between PA and depressive symptoms when pain is included in the model would suggest that pain mediates the relationship between these two variables. In order to test for moderation, an interaction term (PA by pain) was included in the same model with PA, pain and relevant covariates. Evidence of a moderating effect of pain on the relationship between PA and depressive symptoms would be represented by a significant *p* value for the interaction term(s).The secondary outcome variable was health-care utilisation. A multinomial regression assessed the impact of PA, pain and depressive symptoms on the frequency of health-care utilisation over the last 12 months with ‘No GP visits’ as the reference category. Relative risk ratios were used as the measure of effect sizes.

## Results

### Demographics

The total number of participants included in the analysis of TILDA was 8163. Participants ranged in age from 50 to 80+ years with more than half the sample being aged between 50 and 64 years (58.4%). More than half of the participants were women (52%), while more than two-thirds were currently married or living as married (67.9%). A smaller proportion was widowed (15.9%). In terms of social class, as determined by occupation, 31% of older adults were currently unemployed, out of work through long-term illness or looking after a home or family. The next largest proportion of respondents was drawn from social classes 3 and 4 (20.8%) which includes lower professional and non-manual positions. Approximately one third of the participants were educated to secondary level or higher, while almost 4 in every 10 had either none or only primary school education (38.2%). These findings are presented in Table 1.

**Table 1.** Demographics and health and health behavioural characteristics of TILDA participants (*N* = 8163) including chi-square analyses.

Variable name	Total	NDS	DS	*χ*^2^	*p* Value
	*N* (%)	*N* (%)	*n* (%)		(chi-square test)
**Demographics**					
*Gender*					
Male	3740 (48)	3347 (43)	341 (5)		
Female	4423 (52)	3694 (44)	651 (8)	57.8	<.001
*Marital status*					
Never married	790 (9.7)	651 (8)	125 (2)		
Married or cohabiting	5631 (67.9)	5011 (60)	550 (7)		
Separated or divorced	551 (6.6)	413 (5)	124 (2)		
Widowed	1191 (15.9)	966 (13)	193 (3)	36.2	<.001
*Age (years)*					
50–54	1622 (19.7)	1372 (17)	233 (3)		
55–64	3042 (38.7)	2617 (34)	385 (5)		
65–74	2159 (23.4)	1888 (21)	229 (3)		
75+	1340 (18.2)	1164 (15)	145 (2)	2.7	.046
*Social class*					
Social class 1–2	1799 (17)	1643 (16)	139 (1)		
Social class 3–4	1679 (20.8)	1491 (19)	163 (2)		
Social class 5–6	1043 (14.1)	891 (12)	134 (2)		
Unemployed/not applicable	2323 (31)	1812 (25)	462 (6)		
Unknown/refused	795 (9.7)	715 (9)	66 (<1)		
Farmers	523 (7.4)	488 (7)	28 (<1)	34.8	<.001
*Education*					
No education or primary only	2501 (38.2)	2057 (32)	388 (6		
Some second-level education	1900 (25.2)	1664 (22)	215 (3)		
Leaving Certificate or higher	3758 (36.6)	3317 (33)	389 (4)	17.9	<.001
**Health and health behaviours**					
*Level of PA*					
Low	2591 (33.1)	2095 (27)	440 (6)		
Moderate	2780 (33.5)	2437 (30)	310 (4)		
High	2713 (33.4)	2442 (30)	234 (3)	44.9	<.001
*Experience of pain*					
None or mild pain	6101 (74)	5518 (68)	503 (6)		
Moderate pain	1345 (16.7)	1054 (13)	266 (3)		
Severe pain	712 (9.4)	464 (6)	223 (3)	181.7	<.001
*GP visit in the last 12 months*					
Yes	7142 (87.5)	6111 (76)	914 (12)		
No	1021 (12.5)	930 (12)	78 (<1)	23.0	<.001
*Self-rated health*					
Poor	417 (5.5)	214 (3)	180 (3)		
Fair	1482 (19.4)	1139 (15)	307 (4)		
Good to excellent	6263 (75.1)	5688 (69)	504 (6)	291.0	<.001
*Smoking status*					
Never smoked	3561 (42.9)	3164 (39)	349 (4)		
Ex-smoker	3113 (37.7)	2722 (33)	345 (5)		
Current smoker	1488 (19.3)	1154 (15)	298 (4)	45.8	<.001

DS,  depressive symptoms; NDS, no depressive symptoms; *χ* ^2^, chi-square test statistic.

Note: Depressive symptoms were calculated by the Centre for Epidemiological Studies – Depression scale (CES-D). A binary outcome variable (*z*-score) classified all participants who scored 1 SD or more above the mean as ‘depressive symptoms’, and all remaining participants as ‘no depressive symptoms’.

### Depressive symptoms, health behaviours and health-care utilisation

The vast majority of participants (91.4%) were not depressed (i.e. they had not scored above the threshold cut-off points of ≤16 on the CES-D). Current depression was recorded in 8.5% of the TILDA sample. In terms of PA, the sample was divided almost equally across the three levels – low levels of PA (33.1%), moderate levels of PA (33.5%) and high levels of PA (33.4%). Approximately three quarters of older adults reported none or only mild pain in the past week (74%). Proportions of older adults experiencing moderate pain were 16.7%. Almost 9 in 10 TILDA participants (87.5%) reported having visited a GP in the 12 months prior to the study. The median number of GP visits per older adult was three (59.2%) with a range for the sample of 0–25 visits in the last 12 months. Three quarters of participants (75.1%) rated their overall health as good, very good or excellent. As presented in Table 1, the proportion of participants rating their health as poor was just 5.5%. Almost one in five (19.4%) older adults felt their health was fair. The overall rate of current smoking was 19.3% in TILDA. Approximately 43% (42.9%) had never smoked and a similar proportion were former smokers (37.7%). Significant differences were found across all variables between participants with and without depressive symptoms (Table 1).

### Mediation and moderation analyses

The results from the mediation analyses are presented below in Table 2. As described previously (*z*-score), binary variables, classified those who scored 1 SD or more above the mean value as ‘depressive symptoms’, and those who did not as ‘no depressive symptoms’. Both level of PA and experience of pain both independently influenced the likelihood of current depressive symptoms and these effects were seen at all levels of these variables. For example, participants engaged in moderate and high levels of PA were significantly less likely to have depressive symptoms compared to those engaged in low levels of PA. Both moderate and severe levels of recent pain were also associated with an increased likelihood of current depressive symptoms. When both the level of PA and experience of pain were entered into the same model, there is virtually no effect on the RR for PA. This suggests that pain does not mediate the relationship between PA and depressive symptoms.

**Table 2.** Poisson regression testing prediction of depressive symptoms (scoring 1 SD or above) by the level of PA and experience of pain for TILDA cohort.

Variable name	RR (95% CI)	*p* Value
**Model 1 (*n* = 7953)**		
*Level of PA*		
Low	1	
Medium	0.85 (0.74–0.97)	.016
High	0.76 (0.64–0.91)	.003
**Model 2 (*n* = 8022)**		
*Experience of pain*		
None or mild pain	1	
Moderate pain	1.7 (1.5–1.9)	<.001
Severe pain	1.8 (1.5–2.2)	<.001
**Model 3 (*n* = 7948)**		
*Level of PA*		
Low	1	
Medium	0.88 (0.77–1.0)	.061
High	0.78 (0.65–0.93)	.005
*Experience of pain*		
None or mild pain	1	
Moderate pain	1.7 (1.5–1.9)	<.001
Severe pain	1.8 (1.5–2.2)	<.001

CI, confidence interval.

Note: All models adjusted for gender, marital status, age, social class, education, self-rated health, smoking status and number of GP visits in last 12 months.

With the absence of any mediating role of pain in the relationship between PA and depressive symptoms, further models were run to determine if pain had any moderating role in this relationship (please see Table 5). First the proportion of participants with pain and depressive symptoms (as indicated by scoring 1 SD or more above the mean on the standardised CES-D) are presented in Table 3.

**Table 3.** Proportions of TILDA participants with and without depressive symptoms across the three levels of pain.

	Experience of pain		
	None or mild	Moderate	Severe
	*n* (%)	*n* (%)	*n* (%)
*Depressive symptoms*			
No	5158 (91)	1054 (79)	464 (67)
Yes	503 (9)	266 (21)	223 (33)

Note: Depressive symptoms variable calculated using CES-D *z*-scores classifying all participants 1 SD or more above the mean as experiencing ‘depressive symptoms’.

As seen (in Table 3) the presence of depressive symptoms followed an expected pattern with proportions of those with depressive symptoms increasing across levels of pain. This pattern is also evident in Table 4, where the mean depression scores, as measured by the continuous CES-D measure, across levels of PA by levels of pain are presented.

**Table 4.** Mean scores on the measure for depression across the three levels of PA and pain for TILDA participants.

	Level of PA					
	Low		Moderate		High	
	*n*	Mean (SD)	*n*	Mean (SD)	*n*	Mean (SD)
*Experience of pain*						
None or mild	1685	5.8 (6.6)	2149	4.6 (5.8)	2128	4.1 (5.6)
Moderate	495	8.8 (8.6)	427	8.0 (8.2)	387	6.7 (7.4)
Severe	353	12.3 (10.8)	170	11.7 (10.3)	159	8.9 (9.8)

Note: CES-D threshold cut-off of ≥16 indicative of depression.

Furthermore, the CES-D mean scores do not indicate any exponential changes, and thereby suggest that moderating effects may be absent.

The results from the moderation analysis are presented in Table 5. All levels of PA and pain were independently correlated with the presence or absence of depressive symptoms. Furthermore, all interaction terms were insignificant suggesting that pain does not have a moderating role in the association between PA and depressive symptoms in the TILDA cohort.

**Table 5.** Moderation analysis, using Poisson regression, testing prediction of depressive symptoms (scoring 1 SD or more) by the level of PA and experience of pain and interaction terms for TILDA cohort (*n* = 7948).

Variable name	RR (95% CI)	*p* value
*Level of PA*		
Low	1	
Medium	0.76 (0.63–0.93)	.006
High	0.73 (0.58–0.91)	.005
*Experience of pain*		
None or mild pain	1	
Moderate pain	1.5 (1.2–1.8)	<.001
Severe pain	1.6 (1.3–2.0)	<.001
*Interaction terms*		
Low PA × none or mild pain	1	
Moderate PA × moderate pain	1.3 (0.99–1.8)	.058
Moderate PA × severe pain	1.3 (0.96–1.9)	.087
High PA × moderate pain	1.1 (0.80–1.6)	.515
High PA × severe pain	1.1 (0.77–1.7)	.489

CI, confidence interval.

Note: All models adjusted for gender, marital status, age, social class, education, self-rated health, smoking status, and number of GP visits in last 12 months.

### Health-care utilisation analysis

As seen in Table 6, TILDA participants with depressive symptoms were found to be at an increasing risk of more frequent GP visits in the last 12 months compared to those with no depressive symptoms. For example, respondents with depressive symptoms had a 75% higher risk of having five or more GP visits compared to those with no depressive symptoms. The results clearly indicate that as levels of PA increase, the likelihood of an increasing number of GP visits significantly reduces, but only for those engaged in high levels of regular PA. Therefore, moderate levels of PA appeared to have no significant impact on the likelihood of reporting a reduced number of GP visits (in comparison to those with low PA). However, those engaged in high levels of PA were less likely to have attended their GP when compared to those who had low PA levels, with estimates ranging from 18% to 45% reduction in the risk of attending. As expected, increasing levels of pain were also associated with an increased risk of a higher number of GP visits. This was especially true for those reporting at least three (or more) GP visits in the last 12 months. For example, those with moderate levels of pain were at an almost three times significantly higher risk of five or more GP visits compared to those with no pain. This risk increased to almost three and a half times for those with severe pain.

**Table 6.** Impact of depressive symptoms, PA and pain on GP visits in last 12 months for TILDA participants.

Variable name	1–2 GP visits		3–4 GP visits		5+ GP visits	
	RRR (95% CI)	*p* value	RRR (95% CI)	*p* value	RRR (95% CI)	*p* value
*Depressive symptoms*						
Yes	0.925 (0.689–1.24)	.603	1.38 (1.02–1.88)	.039	1.75 (1.27–2.40)	.001
*Level of PA*						
Low	1		1		1	
Medium	0.947 (0.774–1.16)	.596	0.929 (0.752–1.15)	.495	0.920 (0.737–1.15)	.461
High	0.815 (0.669–0.991)	.041	0.698 (0.566–0.860)	.001	0.545 (0.438–0.678)	<.001
*Experience of pain*						
None or mild pain	1		1		1	
Moderate pain	1.23 (0.958–1.57)	.106	1.70 (1.31–2.21)	<.001	2.91 (2.24–3.78)	<.001
Severe pain	1.13 (0.737–1.73)	.575	2.05 (1.34–3.15)	.001	3.42 (2.26–5.18)	<.001

CI, confidence interval; RRR, relative risk ratio.

Note: Model adjusted for gender, marital status, age, social class, education, self-rated health and smoking status. Reference category: No GP visits.

## Discussion

The aim of this study was to investigate whether pain mediates or moderates the relationship between PA and depressive symptoms in Irish adults aged 50 years or more. The impact of these factors on health-care utilisation was also examined. Based on our analyses, pain did not have a mediating or moderating role in the relationship between depressive symptoms and PA in older adults. As anticipated, participants with depressive symptoms and those with pain were more likely to have an increased number of GP visits in the last 12 months. There was also some evidence for the protective effect of PA in terms of decreased health-care utilisation.

Approximately, one-third (33.1%) of older adults in TILDA reported low levels of PA. Morgan et al. (2011), who also examined PA levels in a sample of Irish older adults (from the RoI, 2005–2006, and Northern Ireland, 2007), reported slightly higher levels in this category at 35.6% (RoI participants only). This suggests a slight shift in the amount of older adults engaging in increasing levels of regular PA. For example, 15.5% older RoI adults in the Morgan et al. (2011) study reported high levels of PA compared to 33.4% of the participants in this study. In summary, the proportion of older adults in RoI engaging in high levels of PA appears to have increased. This could be as a result of initiatives in this region in the time between studies or a reflection of a more health-conscious cohort of ageing adults. Experience of recent pain was another key variable investigated. Twenty-six per cent of TILDA participants reported some level of recent pain. While the duration of pain (i.e. three months or longer would indicate chronic pain) is not considered here, comparable levels of chronic pain in the Irish population have been reported at 35.5% (Raferty et al., 2011) and at 45% in a community survey conducted in England (*N* = 4172) (Carnes, 2011).

In terms of the main aims of the study, no mediating or moderating effects of pain on the association between PA and depressive symptoms were seen. Adding pain to the models did not attenuate the association between PA and depressive symptoms. Thus, higher levels of PA were protective against depressive symptoms, irrespective of the levels of pain an older adult reports. Similarly, there was no synergistic interaction between pain and PA in the relationship with depressive symptoms. In other words, while incremental associations were seen between PA and pain and depressive symptoms, as expected, the combination of PA and pain did not provide any multiplicative effects over and beyond each variable alone. Thus, pain levels did not moderate the association between PA and depressive symptoms. This is in contrast to previous findings that reported a partial mediating effect of PA on the relationship between pain and depressive symptoms (Sabiston et al., 2012) and the moderating effect of depressive symptoms on PA demonstrated at all levels of pain reported by Mossey et al. (2000). Given that our analyses were conducted on population-based data and not small community-based samples, it is likely a robust conclusion that pain does not mediate or moderate the relationship between PA and depressive symptoms, and that previously reported results could be chance findings.

Finally, in relation to health-care utilisation, as expected, higher levels of depressive symptoms were associated with an increased number of GP visits. This also supports previous findings in the literature (Herrman et al., 2002; Luber et al., 2001). The most consistent finding was that increasing pain levels were associated with increased GP visits. While prevalence estimates vary, pain gradually increases as we age (Gibson & Lussier, 2012). The associated burden on health services, as demonstrated here and elsewhere (Blyth et al., 2004), necessitates effective pain management techniques that eases this demand and adequately relieves unnecessary suffering in older cohorts.

This study makes an important, clinically relevant contribution to active ageing research. To our knowledge, no other population-based approach has investigated the mediating or moderating effect of pain on PA or depressive symptoms in older adults. Overall, our findings suggest that pain does not play a mediating or moderating role in the relationship between PA and depressive symptoms. Instead, our analyses found that both PA and pain were independently associated with depressive symptoms in Irish older adults. Therefore, our findings would support ongoing initiatives in this area. One example in Ireland is the *Go for Life* initiative (http://ageandopportunity.ie/node/40), which has been designed and implemented with a view to encouraging and supporting older people to engage in more PA, thereby improving their mental and physical health. Our results also show that clinicians and health service providers should continue to consider and promote PA as a treatment for depression, irrespective of the pain levels of their patients. Although PA and pain are clearly inter-related, there is no evidence of synergistic effects. Therefore, treatment plans or interventions need to consider both of these factors independently.

These findings will be relevant for a range of health-care professionals, health promotion, policy-makers and service providers and provide important insights into how the physical and mental health of respondents may be improved. The importance of the treatment of depression in older people has been highlighted repeatedly (Chapman & Perry, 2008) and the protective effects of PA are evidenced in older people. These findings also inform public health and policy approaches for active and healthy ageing (e.g. public transport to increase PA, the need for effective pain management, etc.). Given the ageing population both here and abroad, the increasing investment in initiatives and strategies aimed at improving mental and physical health in older people is an intuitive step and potentially cost-effective in the long-term.

The authors acknowledge that this study has a number of limitations. One limitation is the difficulty in reliably measuring PA in surveys, which has been noted previously (Morgan et al., 2011). Second, the use of the standardised scores of the depressive symptom measures over the threshold scores may have influenced our results, however; it also facilitates comparisons across other population surveys that have used different measures of depression. Third, the increased risk of higher GP visits in participants with depressive symptoms and higher levels of pain may be a factor of more frequent monitoring as necessitated by chronic conditions such as these. Similar to other population studies, this study is also limited by the fact that the survey was cross-sectional and utilised self-report. Cross-sectional data present the likelihood of associations between variables but do not allow the inference of causation. Self-report data have inherent biases associated with it such as social desirability and recall bias (Rubenstein, Schairer, Wieland, & Kane, 1984). For example, one study found that older participants were more likely to over-report their levels of PA (although under-reporting errors were noted too) (Heesch, van Uffelen, Hill, & Brown, 2010). Irrespective of causality, the evidence-based bidirectional relationship between PA and depressive symptoms (Roshanaei-Moghaddam, Katon, & Russo, 2009; Teychenne, Ball, & Salmon, 2008) and pain and depressive symptoms (Chou, 2007; Mossey et al., 2000) suggests that interventions should incorporate strategies relevant to both of these factors.

In conclusion, these findings suggest that pain does not have a mediating or moderating role in the association between depressive symptoms and PA. Importantly, they suggest that clinicians can continue to recommend PA for management of depression, irrespective of an individual's pain levels (assuming that pain is not directly preventing PA). As well as improving mental and physical well-being, engagement in PA, particularly at high levels, will also reduce the burden on health-care services.
